# Dates fruits effects on blood glucose among patients with diabetes mellitus: A review and meta-analysis

**DOI:** 10.12669/pjms.37.4.4112

**Published:** 2021

**Authors:** Hyder Osman Mirghani

**Affiliations:** Hyder Osman Mirghani, MD. Associate Professor, Department of Internal Medicine, Faculty of Medicine, University of Tabuk, Tabuk, Saudi Arabia

**Keywords:** Dates fruit, Fasting plasma glucose, HbA1_c_, Postprandial plasma glucose

## Abstract

**Background & Objective:**

Dates fruit is known for its great nutritional value and two to three servings of dates fruit/day are beneficial for patients with diabetes. However, some may advice against this cheap and widely available fruit consumption. Besides, 12% of the population Worldwide are either suffering or are at risk of developing diabetes, but no previous meta-analysis has assessed this important issue. Thus, the study aimed to investigate the effects of date’s fruit on glycemia among patients with diabetes.

**Methods:**

A systematic literature search was conducted in PubMed, Medline, EBSCO, Cochrane, and Google Scholar databases for trials published in English from the first published article up to December 2020. The following keywords were used: ^“^dates fruit and glycemic control^”^, “dates fruit and blood glucose^”^, ^“^dates fruit and HbA1_c_^”^ without limitations regarding the date of publication.

**Results:**

Out of the 942 references identified, only 10 cohorts from five full texts were included, a reduction of Fasting plasma glucose (FPG), odd ratio, -24.79, 95% CI=-34.75, -14.83 P =0.002. *I^2^* for heterogeneity=79%, P <0.00001 and postprandial plasma glucose (PPPG), odd ratio -28.19, 95% *CI=*-60.66-4.29, *P* =<0.0001. *I^2^* for heterogeneity=92%, *P*=0.09) was observed. While the effect on HbA1_c_ was neutral, odd ratio, -.20, 95% CI=-.46 -.06, P=0.13. I^2^ for heterogeneity=0. %, P=0.55.

**Conclusion:**

Dates fruit was beneficial regarding glycemic control among patients with diabetes, physician may not need to restrict its use among patients with diabetes. The small number of the included studies and the heterogeneity observed in PPPG and FPG sub-analysis limited the current results. Further trials assessing the glycemic indices of various types of dates fruit are needed.

## INTRODUCTION

The date palm (Phoenix dactylifera L.) is among the oldest plants worldwide. The plant is closely linked to the lives of people in the Middle East and Arab Countries since ancient times. Dates have been used as food for more than six thousand years. In addition to its nutritional value as a good energy source, dates might prevent many diseases due to its anti-inflammatory, anti-bacterial, and anti-oxidant actions.^1^,^2^ The various health and nutritional benefits was described in many religions and cultures.^3^-^5^ Animal studies showed the anti-hyperglycemic effects of Aseel dates.^5^ Due to its high content of 13 phenolic compounds, dates fruit showed specific inhibitor of α-glucosidase and was found to reduce plasma sugar more than Acarbose after 30 minutes of ingestion.^6^ Furthermore, it was shown to be effective for the prevention of Type-2 diabetes, and many other health problems.^7^ Ajwa and Sukkari of the Kingdom of Saudi Arabia showed antidiabetic properties among diabetic rats^8^ and randomized controlled trials conducted on healthy humans showed that dried dates reduce the glycemic response of white bread and Tamersit type reduced blood glucose.^9^,^10^ Further animals and experimental studies concluded that dates fruit significantly reduce blood glucose more than acarbose (a glucosidase inhibitor used for the treatment of diabetes mellitus).^8^-^11^,^12^

Regarding microvascular diabetic complications, a preventive role in diabetic neuropathy was reported by Zangiabadi et al.^13^ It is interesting to note that, despite its high potassium contents, date consumption (ten fruits) did not significantly increase the potassium levels among patients with renal failure (diabetes mellitus is the leading cause).^14^ Dietary measures play a major role in the management of metabolic diseases and the high consumption of dates fruit in certain cultures raises the concern whether patients with diabetes mellitus should eat dates fruits. Despite the importance of dates fruit as a good source of energy, few studies have assessed its effects on glycemic control. Thus, we conducted this meta-analysis to assess the effects of date consumption on plasma sugar and HbA1_c_ among patients suffering from diabetes.

## METHODS

### Eligibility criteria according to PICOS

We included trials on humans investigating the effects of dates fruit on blood glucose and glycated hemoglobin. Studies on animals and studies with methodologies other than trials were excluded. Participants were adults (more than 18 years of age) with diabetes mellitus, children and pregnant women were not included. The studies were included if they assessed one of the following outcomes:

- The effects of dates fruit consumption on the blood glucose.
- Effects of dated fruit on HbA1_c._
- Effects of dated fruit on post-prandial blood glucose.
- The glycemic index of dates fruits.

### Information sources and search methods

**Fig.1:**
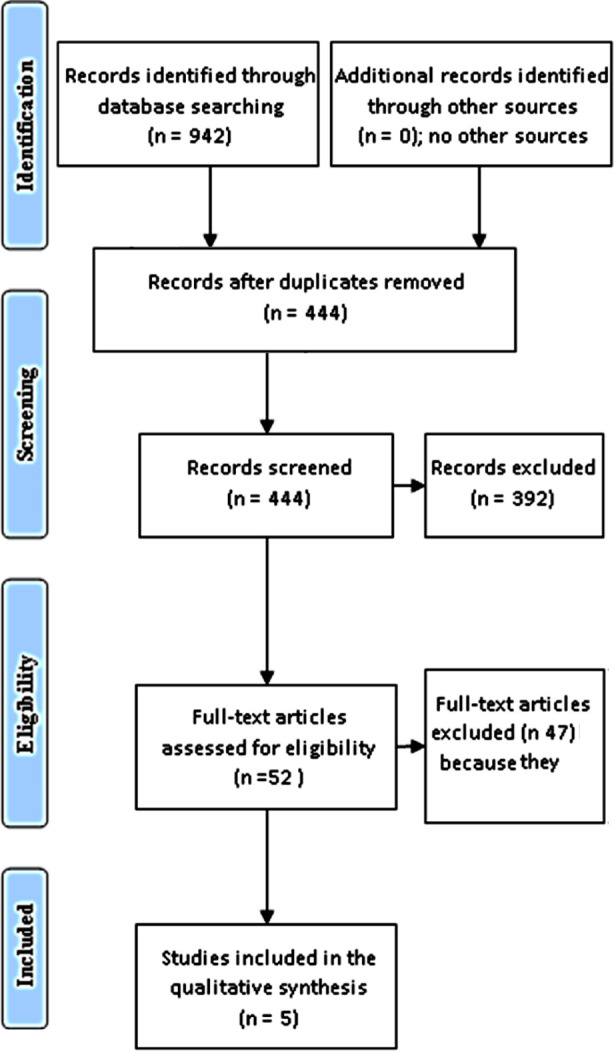
Flow diagram through the different phases of the systematic review (PRISMA flowchart).^15^

A systematic literature search was conducted in PubMed (132 articles), EBSCO (186 articles), Cochrane library (124 articles), and the first 500 articles of Google Scholar database. No limitation was applied to dates of publication (from the first published article up to December 2020), and only articles published in English were included. The following keywords were used: ^“^dates fruit and glycemic control^”^, “dates fruit and blood glucose^”^, ^“^dates fruit and HbA1_c_^”^, the same was used replacing dates fruits with Palm dates, Ajwa, Khalas, or Pheonix dactilefera. Regarding the different species of Palm dates, the search engine was set to Pheonix dactilefera, others including Phoenix atlantica, and Phoenix caespitosa were not. The author searched the results manually, the abstracts and full texts were screened for relevant articles, and the references of the included studies were also searched for additional information. Out of the 942 references identified, only 52 full text were screened and the final analysis included 10 cohorts from five trials. The remaining text were excluded because of no full text were available in some old studies, text of some studies were not freely available (needed payment) , or the low quality. The data fulfilling the inclusion and exclusion criteria were then entered in a data extraction sheet. The sheet included the author’s name, year of publication, study type, the number of patients included, the type, amount, and duration of dates consumption, glycemic index, blood glucose, and the glycated hemoglobin before and after date’s fruits intake by the same participant or measures of glycemia before and after date’s fruits consumption among participants and control subjects. The PRISMA Chart was used for reporting the phases of the systematic review.^15^ Fig.1.

### The risk of bias assessment in the included studies

The modified Cochrane score for assessment of bias was used to assess the included studies quality.^16^

### Statistical analysis

The Revman system (version, 5.4) for reviews was used for data analysis, the included studies were manually entered, the item interventional studies were chosen, and continuous data were entered with 95% confidence interval, mean difference, and heterogeneity estimation. The fixed effect was chosen for HbA1c, while the random effect was applied for PPPG and FPG due to the high heterogeneity observed (>50%). A *P* <0.05 was considered significant. The sensitivity was estimated by the funnel plot. The information regarding the used date fruits was depicted in (Table-I).

**Table-I:** The included trials risk of bias as assessed by the modified Revised Cochrane risk of bias tool for randomized trials.^16^

	Selection	Performance	Attrition	Reporting	Other
Foshati et al.2015^17^	High	High	Low	Low	Low
Alalwan et al. 2020^18^	High	Low	Low	Low	Low
Algheshairy RM. 2018^19^	High	High	Low	Low	Low
Ali et al. 2018^20^	Low	Low	Low	Low	Low
Forghani et al.^21^	High	High	Low	Low	Low

## RESULTS

**Fig.2:**
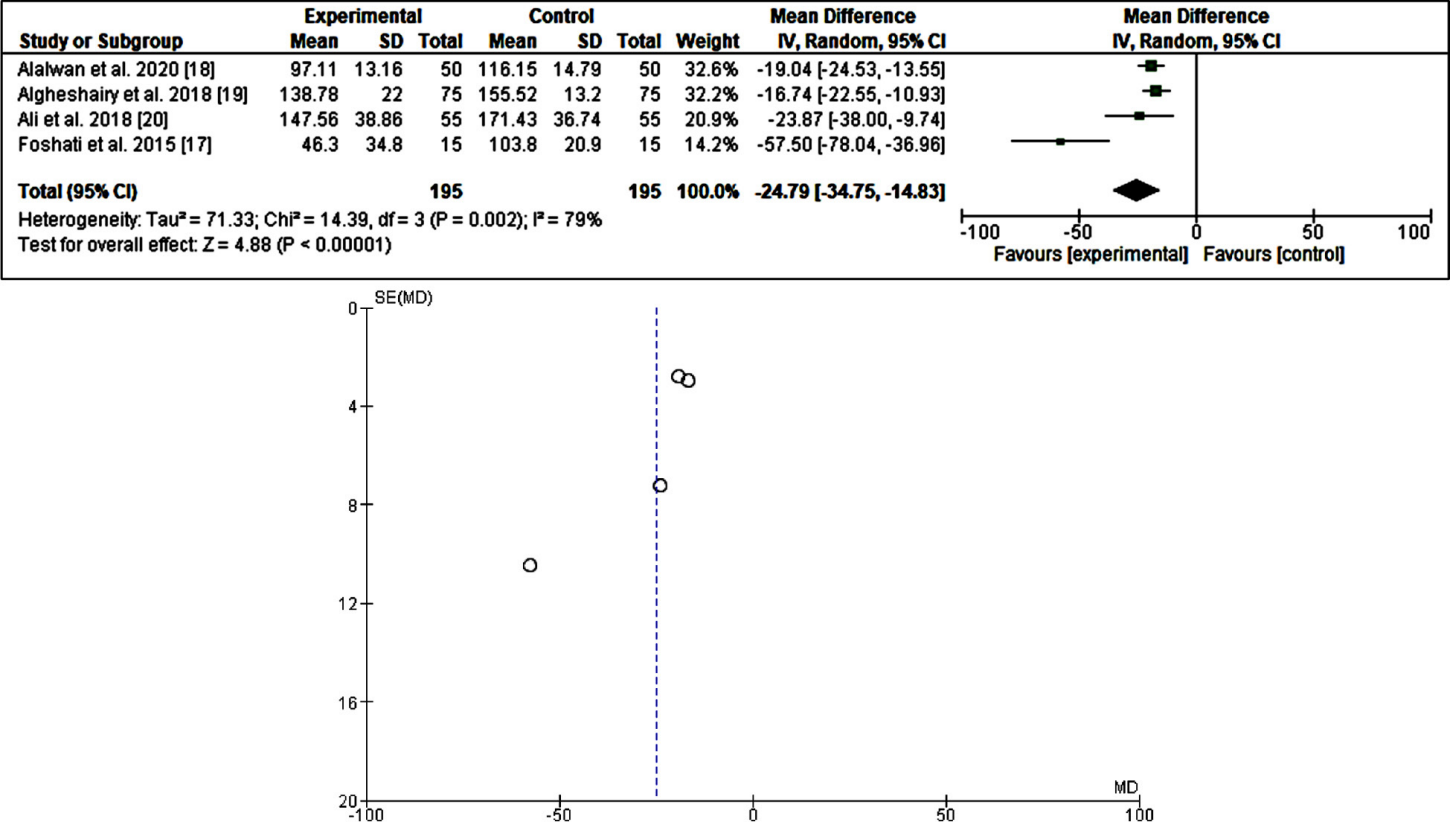
The effects of dates fruit (Phoenix dactylifera L.) on fasting blood glucose.

**Fig.3:**
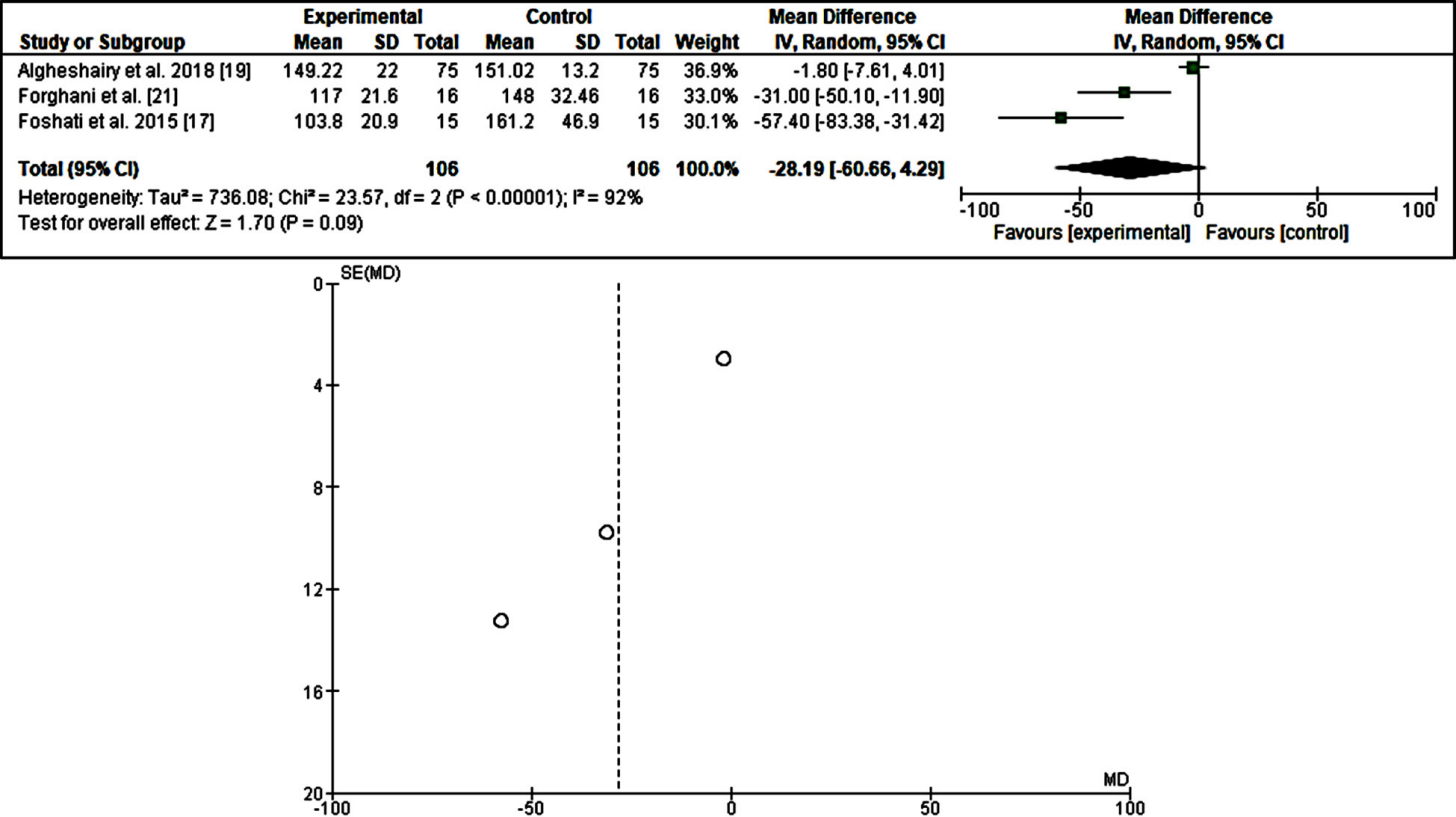
The effects of dates fruits on postprandial blood glucose.

**Fig.4:**
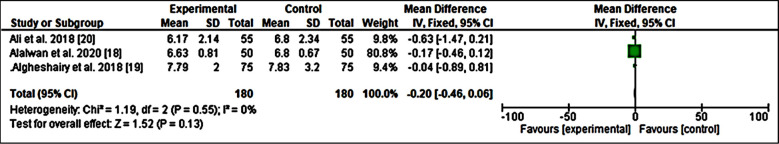
The effects of dates fruit (Phoenix dactylifera L.) on HbA1c.

Out of the 942 articles identified, 52 full texts were screened, and only five full articles fulfilled the inclusion and exclusion criteria, all were published in Asia. The included studies approached 390 adult participants with diabetes mellitus, the duration of dates consumption ranged from one day to 12 weeks. In the present meta-analysis, all the included studies showed a reduction of fasting blood glucose 17-20, odd ratio, -24.79, 95% CI=-34.75, -14.83 P =0.002. *I^2^* for heterogeneity=79%, P <0.00001, three studies were investigated the effects of dates fruit consumption on post-prandial blood glucose, all the studies showed a reducing effect^17^,^19^,^21^, odd ratio, -28.19, 95% *CI=*-60.66-4.29, *P* =<0.0001. *I^2^* for heterogeneity=92%, *P*=0.09. Regarding the HbA1_c_, the three included studies showed a neutral effect^18^-^20^, odd ratio,-0.20, 95% *CI=*-.46 -.06, *P*=0.13. *I^2^* for heterogeneity=0. %, *P*=0.55. (Table-II & Table-III) and (Fig. 2-4).

**Table-II:** The dates fruit types, amount, and duration of use among the participants.

Author	Type of date	Amount	Duration
Foshati et al.^17^	Not stated	19 grams in tea 150 ml of tea	Once
Alalwan et al.^18^	Khudary (local, Bahrain)	3 dates/day	6 weeks
Algheshairy et al.^19^	Ajwa (local, Saudi Arabia)	One fruit/day	12 weeks
Ali et al. 20	Dates vinegar	20 ml/day	10 weeks
Forghani et al.^21^	Dates mixed with food	50% of carbohydrates replaced with dates	One day

**Table-III:** Glycemic indices of various types of date’s fruit in some of top 10 producers.

Type and country of dates	Glycemic index
***Saudi Arabia (ref.32)***	
Sellag	74.6 ±10.1
Maktoomi	71.0 ±11.1
Shaqra	42.8 ±5.5
Sukkary	43.4 ±4.7
Sag’ai	44.6±6.0
***United Arab Emirates (ref. 30, 35)***	
Khalas	Ranged from 35.5-55.1±7.7
Fardh	Ranged from 54.0 ± 6.1-57.7
Barhi	49.7
Bo ma’an	Ranged from 35.5-46.3 ± 7.1
Lulu	Ranged from 35.5-46.3 ± 7.1
Dabbas	53.5 ± 8.649.1 ± 3.6 49.1 ± 3.6
***Oman (ref. 28)***	
Khalas	47.6
Fardh	57.7
***Egypt (ref. 31)***	
Partamoda	65.92 ± 1.62
Malakabi	69.04 ± 1.72
Saadi	67.42 ± 2.25
Zaglool	30.36 ± 0.72
Samani	34.69b ± 1.24

**Table-IV:** Details of the studies included in the meta-analysis.

Author	Year	Country	Study type	Patients	Results
Foshati et al.^17^	2015	Iran	Cross-over trial	15	Fasting and postprandial plasma glucose reduction
Alalwan et al.^18^	2020	Bahrain	Randomized controlled	100	Reduction of FPG and HbA1_c_
Algheshairy et al.^19^	2018	Saudi Arabia	Randomized controlled trial	75	Reduction of FPG, postprandial, and HbA1_c_
Ali et al.^20^	2018	Pakistan	Randomized double-blinded trial	55	Reduction of FPG and HbA1_c_
Forghani et al.^21^	2003	Iran	Non-randomized controlled trial	16	Postprandial plasma glucose reduction

## DISCUSSION

The current data showed that dates fruits reduce fasting plasma glucose with significant statistical difference^17^-^20^ (odd ratio, -24.79, 95% CI=-34.75, -14.83 P =0.002. *I^2^* for heterogeneity=79%, P <0.00001). Postprandial plasma glucose was also reduced 17, 19, 21 but with significant heterogeniety (17,19,21, odd ratio, -28.19, 95% *CI=*-60.66-4.29, *P* =<0.0001. *I^2^* for heterogeneity=92%, *P*=0.09), with neutral effects on the glycated hemoglobin^18^-^20^ (odd ratio,-0.20, 95% *CI=*-.46 -0.06, *P*=.13. *I^2^* for heterogeneity=0.%, *P*=0.55.). The above results imply that the fruits of the date are beneficial in terms of glycemia for those suffering from diabetes mellitus. Dates fruits are rich in phytochemicals including sugar, vitamins, and minerals and is good nutritional source valuable for both healthy individuals and patients with diabetes mellitus in particular those taking metformin (a rare cause of vitamin B12 deficiency).^22^,^23^ In addition, dates fruits might lower the plasma sugar and diabetic retinopathy due to its high contents of minerals including magnesium.^24^,^25^ The discrepancy observed in dates effects on glycemia might be explained by the fact that the glycated hemoglobin varied widely across age, race, and associated morbidities.^26^,^27^ Besides, the small number and heterogeneity observed in the included studies may substantially affect the results. Also, the dates varied greatly by country of origin, methods of cultivation, and the type of the fruit.^28^ The glycemic index and glycemic load are other determinants of date fruit effects on glycemia.

### The glycemic index of some types of dates fruit

Glycemic indices are used to classify foods in particular those with high carbohydrates by measuring the effects of a meal on postprandial glucose levels. The rate and the length of time that plasma glucose remains elevated after a meal can modulate various diseases by affecting the magnitude of metabolic pathways and hormones.^1^ In the current review, AlGeffari and colleagues^1^assessed the glycemic indices of different dates fruits in Qassim, Saudi Arabia, and found a glycemic index ranging from 42.8 to74.6. No significant differences were observed between the different types (highest in Sellaj and Maktoomi, and lowest in Shaqra, Sukkary, and Sag’ ai). However, the glycemic load differ significantly and was lowest in Ajwah and Shaqra. The findings of the same glycemic index in different types of dates fruits was supported by Ali et al.^29^ from Oman. Further studies^30^ from the United Arab Emirates compared different classes (Tamer stage) among people with and without Type-2 diabetes and found no differences in glycemic index between different groups. On the other hands, Miller et al.^31^ assessed the glycemic index of three different classes of dates fruit from UAE and found that boma’an had the lowest glycemic index than khalas and barhi with a significant statistical difference. A study conducted in Egypt concluded that Zaglool dates showed the lowest glycemic index, while partamoda had the highest.^32^ In the current review, Al-farsi et al.^33^ observed that Shagra and Ajwa had the lowest glycemic index.

### The glycemic indices of dates fruits when mixed with or compared to some meals:

Alkaabi et al. 34 assessed the effects of Arabic coffee on Khalas glycemic index among healthy individuals and patients with diabetes and found no differences between groups indicating that coffee intake with dates fruit might not affect Khalas glycemic index. It is interesting to note that Ahmed et al. 35 compared Khalas and a modified urban Saudi breakfast and observed a lower glycemic index of Khalas (57.7±8.5 vs. 79). Miller et al.^36^ conducted another study and compared different classes of dates with and without yogurt and concluded that no significant differences in glycemic indices except for rutab vs. commercial tamer dates. Miller and colleagues’ results indicated that dates glycemic index is not affected when mixed with yogurt. In the present review, JARRAR et al. 37 found a low glycemic index of dried *Bisr* and dried *Tamr* dates compared to a standard diet, and Viguiliouk et al.^9^ published a study in Canada and found that dates fruits had a lower glycemic index than white bread.

### The importance of the consideration of both the glycemic index and glycemic load:

Glycemic index is a measure of glycemic response to isoglucidic foods, however, it might not represent the true effects of individual servings of different food, while glycemic load is a measure of the overall effects of standard food portions. Different glycemic indices and loads of different foods are not necessarily reflecting the carbohydrates content. 38 Because of this, the consideration of both the glycemic index and glycemic load is important. AlGeffari et al 1 assessed the glycemic load in some Saudi dates fruits and found that Ajwa and Shaqra had low glycemic loads (8.5 and 9.2 respectively), a high glycemic load was found in Sellag (24). Increased insulin action and decreased glucose absorption were suggested as mechanisms of action for glucose reduction.^39^

### Strength of the study

The strength of the current meta-analysis might be that this is the first one to analyze the effects of dates fruits on glycemia and including randomized trials. We found two narrative reviews focusing on the therapeutic effects of dates fruits.^40^,^41^

### Limitations of the study

The limitations of the study were the small studies included and the high heterogeneity observed regarding blood glucose estimation. Also, being a single author manuscript, the limitation of the search engine to English, and the fact that we did not limit for the period of the study included might increase the bias. Besides, we could not compare the glycemic indices and glycemic loads due to the wide range of dates fruit and the minimal data comparing them against a standard to make bring out a sensible analysis.

## CONCLUSION

Dates fruits were found to reduce fasting plasma glucose and postprandial plasma glucose. A neutral effect on HbA1_c_ was observed. The results regarding glycemic indices of different types of dates fruits are conflicting with some studies showed significant differences and others concluded no difference. The consumption of dates with coffee, and yogurt might not negatively affect its glycemic index. Although it is difficult to draw a conclusion, dates fruit might be a good cheap source of energy and valuable nutrients with positive effects on glycemia among those suffering from diabetes. The present evidence suggested that, dates fruit had a lower glycemic index than some types of meals. Further studies assessing the glycemic loads, indices, and effects on glycemia of different types of dates among the different populations are needed.

### Abbreviations

FPG: fasting plasma glucose, PPPG: postprandial plasma glucose.

### Author’s Contribution:

**HM:** The concept, design, data collection, interpretation, and analysis, manuscript drafting, and critical revision before submission.
